# Faculty development program assists the new faculty in constructing high-quality short answer questions; a quasi-experimental study

**DOI:** 10.1371/journal.pone.0249319

**Published:** 2021-03-29

**Authors:** Hamza Mohammad Abdulghani, Kamran Sattar, Tauseef Ahmad, Ashfaq Akram, Mahmoud Salah Khalil

**Affiliations:** 1 Department of Medical Education, College of Medicine, King Saud University, Riyadh, Saudi Arabia; 2 Department of Medical Education, School of Medical Sciences, Universiti Sains Malaysia, Kelantan, Malaysia; 3 Department of Computer Science and Information Technology, NIMS University, Jaipur, Rajasthan, India; KTH Royal Institute of Technology, SWEDEN

## Abstract

Faculty development programs (FD) prepare the faculty for their educational role and career tasks. We aimed to evaluate the effectiveness of FDP in advancing the quality of short-answer questions (SAQs). This was a quasi-experimental study, comprising 37 new faculty. The SAQs were examined on psychometric analysis and Bloom’s cognitive levels for the two educational blocks of 1st medical year (i.e. Musculoskeletal (MSK) and Renal blocks). We found substantial improvement in the discrimination index values of SAQs prepared after the workshop (p = 0.04). A higher number of SAQs with moderate difficulty and higher discrimination were also observed. Flaws within the post-workshop questions were reduced (3.0%) when compared with pre-workshop (12.5%). The major incline was also reported within Bloom’s cognitive levels when pre-workshop K2 questions (30%) were compared with post-workshop (45.5%) with a p-value = 0.05. The SAQs constructed by the faculty member without participating in FDP are generally of unsatisfactory quality. After the FDP the assessment items of two blocks improved for various parameters of student assessment. The current study advocates that newly joined faculty shall be provided with the FDP to be guided, trained and supported for improving the quality of assessment through SAQs items writing.

## Introduction

The process of faculty development (FD) prepares and enriches the efficiency of faculty for teaching, assessment, research, and other pertinent educational related roles [1]. Moreover, resource material and facilitation development is also achieved which are prerequisites for student centered learning [2]. There is a global consensus on the understanding that assessment drives students’ learning thus, the high-quality assessment has become an indispensable skill in education [3]. An effective teaching assessment structure is an essential element in medical training [4, 5]. As a regular practice, educators themselves develop the test items and or rely on item banks as a source for the assessment. Depending on commercial question banks sometimes lead to the flawed questions whenever and wherever there is a lack of professionally trained staff [6]. Hence, for a valid and reliable assessment, the exam tools and items must be flawless, valid, reliable, and fit for estimating the assorted qualities of expert abilities. Scholars, worldwide agree on the best assessment method to test superficial and deep learning among students [7]. Short-answer questions (SAQs) have undeniably emerged as one of the most often used proficiency test types. Although, SAQs creation and checking are more challenging than some of other forms of questions for instance multiple choice questions (MCQs) [8, 9], yet still are favored and used. The Short Answer Question (SAQ) as a semi-structured, open-ended question format along with a planned, organized and predetermined marking scheme improves assessment’s objectivity. In the medical colleges, well-structured SAQs questions are mostly used to incorporate clinical scenarios. Uses of well-structured exam questions (items) bring the overall quality to the assessment, due to exam items with the validity, higher exam reliability [10].

Well-structured SAQs are capable of testing learners’ higher levels of cognition as well as efficiently help distinguish between a high achiever and low achiever [11, 12]. On the downside of SAQs, like any other assessment item, is the quality which might be compromised due to not adhering to the item construction guidelines by the faculty [11, 13]. To overcome this potential hurdle in getting a high quality assessment item (SAQ), the responsible faculty may be guided and supported to undergo a formal training and experience [14]. Among the available approaches towards the goal of quality item preparation, the faculty development programs (FDPs) stand as a robust method. FDPs offer educational and learning prospects accessible to academic faculty ranging from conferences to formal hands-on workshops targeting the development of assessment items to for medical college courses. However, for a successful FDP the participants require active participation with thoughtful consideration to ensure steadiness for personal development linked with improvement in the teaching, learning and assessment process [15]. Academic staff training for updated knowledge and skills has become an essential entity due to ever changing science and technology with inclusion of innovative teaching methods in our everyday teaching. FDPs offers a multidimensional approach for evidence-based learning methods as well as assessing mediations to keep pace and continue with the improvement in the medical education curriculum [16].

To fulfill the needs mentioned above, the Faculty Development Unit in the medical education department, of the college of medicine, King Saud University (KSU), Saudi Arabia taken an initiative during the academic year 2018–19, and a FD training program comprising of two “full-day” hands-on workshop for development of high quality SAQs were organized. This training program focused on guiding and training of all recently joined teaching faculty at College of Medicine. The focal point of the workshops training program was to guide and train new faculty to develop quality SAQs. Along these lines, the current study aims to evaluate the effectiveness of well-structured FDPs to improve the quality of SAQs items’ writing.

## Methodology

The current study took place at the College of Medicine (COM), King Saud University (KSU) a premier University of Saudi Arabia. The curriculum for bachelors of medicine and bachelor of surgery degree is scattered over five years and divided into 4 phases: Phase1 in college of medicine is called preparatory year (comprising the teaching of main subjects like Physics, Chemistry, Math, English, Biology) Phase 2: first and second medical year known as pre-clinical years with normal and abnormal function and structure of human organ systems, including subjects of basic sciences, intertwined with clinical relevance; Phase 3: the 3r year onwards called clinical-year including main clinical subjects. Whereas, phase 4: comprises of mainly training in the hospital clinics related to various disciplines to achieve the internship clinical requirements.

The study protocol was approved by the institutional review board college of medicine, KS. All participants were provided with a written consent at the start of the study.

### Participants of the study

A general invitation was displayed at various places inside the college, and also the same was advertised through the college’s main website. All newly joined faculty at CoM, KSU belonging to different academic positions in basic science and clinical departments were invited. They comprised of lecturers, assistant professors, associate professors and full professors who started the job at COM, KSU, within last 2 years duration. These belong to basic medical science subjects (all five years) as well as clinical departments (various departments). Their teaching experience ranged from novice (lecturers who has joined COM, KSU as their first job after graduation) to expert (assistant, associate and full professors for whom this was not their first job).

A total of 37 newly joined faculty members participated in the current study. A written consent from all participants was obtained at the beginning of the study. SAQ construction guidelines based on pertinent literature review was developed by the assessment committee members at, Assessment and Evaluation Center (AEC), COM. The new faculty members of the COM were instructed to follow the SAQ construction guidelines for items writing. It was identified by the examination committee that the faculty did not follow and adhere to the uniform principles of the SAQ construction checklist. Hence, the faculty AEC, COM, KSU planned and conducted two full-day hands-on workshops on SAQ items writing. The workshop’s substance included intuitive, informative sessions along with hands-on SAQ item development training toward the start of the academic year 2018–2019.

### Training workshops intercession

AEC has been successfully organizing high standard FDPs in the form of training workshop since the academic year 2012. In a normal routine, the AEC organizes one workshop every year, for various assessment tools e.g. multiple choice questions (MCQs), objective structured practical examination (OSPE), and objective structured clinical examination (OSCE).The post exam analyses of these assessment tools have always shown a good score (satisfactory scores of DF and DI). Whereas, no training workshop was so far conducted targeting SAQs. It was also noted that SAQs’ post exam analysis carried unsatisfactory scores, with a high number of students’ complaints. As a routine practice, the AEC always provides written guidelines including a checklist to all those who are responsible in exam-item preparation. After every exam an extensive feedback to the faculty and students involve in the exam is provided and for this, a comprehensive quality check procedure is in place. This is achieved through a thorough post exam psychometric analysis of all exam tools including, the SAQs. Doing so, it was noted that SAQs have numerous writing flaws (e.g. related to difficulty factor, discrimination index, items non-matched with course objectives etc.), which might be the reason for increased number of complaint from the students. Such a situation helped the examination committee determine that the faculty while preparing the questions are not truly adhered to the recommended and guided uniform best practices. This might pose increased exam related stress to the medical students, and reducing it might help students learn fast [17, 18]. This prompted for organizing a FDP aiming for high quality SAQs. The FDPs were organized for two-day (full-time) and the newly joined faculty members of the COM, KSU. The main objective of these workshop was to train faculty members on how to prepare good quality SAQ for basic science courses. The participants of the workshop were requested to prepared and bring five to six SAQs from their respective specialties to be critically reviewed during the workshop. Theoretical backgrounds were addressed at the beginning of the day along with the revision of the requirements for the SAQs building checklist and agreement was reached with the participants grading the guideline items.

Whenever a dispute related to any guideline statement raised, it was addressed and resolved with majority consensus. On the basis of the accepted list of guidelines for the preparation of SAQs, all pre-workshop SAQs questions (prepared and brought by the participants), were corrected and revised accordingly. On the second day, the participants of workshop were distributed into three to four members in a small group and given the task of making of five new SAQs in their specialties, based on the provided and agreed guideline. Afterward, these SAQs were, discussed, thoughtfully reviewed, altered and rectified with the assent of the participants.

### Post-workshop follow-up

**Fig 1:**
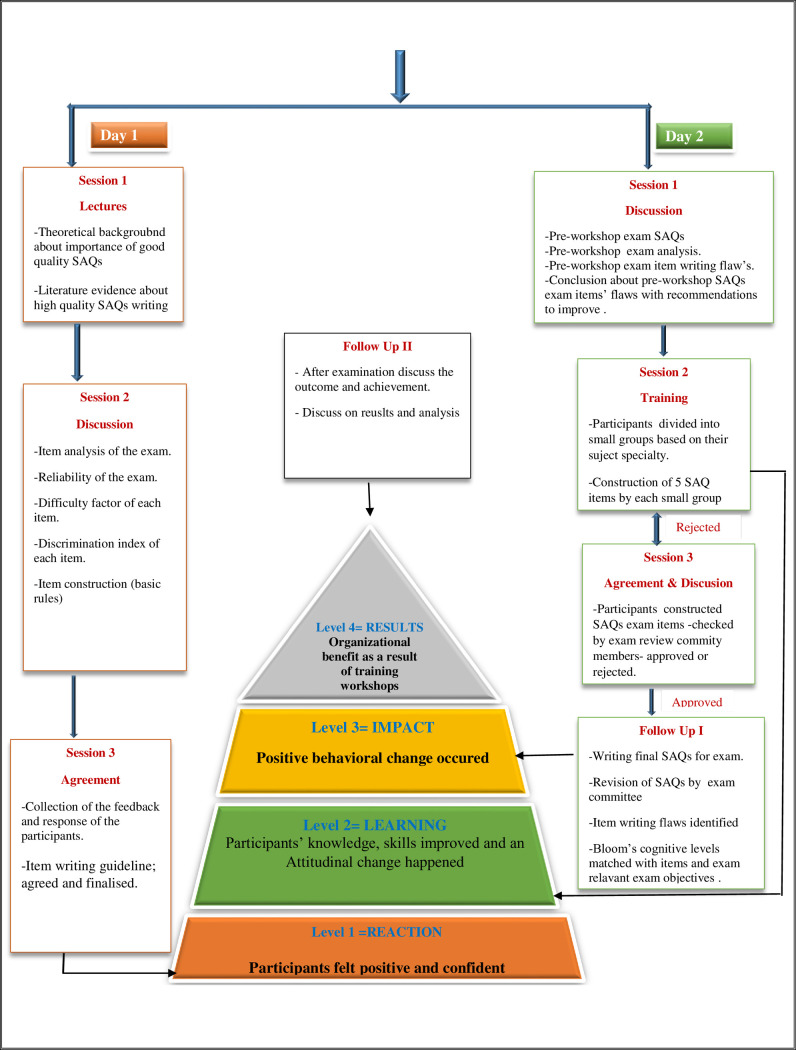
Structure of SAQs items’ writing construction workshop flowchart of the workshop activities in relation to Kirkpatrick’s model.

After the successful completion of FDP training (i.e. workshop), the newly constructed quality SAQs (based on the SAQs construction guidelines. (S1 Appendix) were selected to be used in the assessment for the undergraduate 1st-year final examinations of the two courses (Musculoskeletal and Renal courses). As an initial step for revision of these SAQs respective department committee reviewed based on the subject and block objectives, than AEC exam committee (comprising of experts from various subjects of basic and clinical specialties) has to do a final review based on the guideline and college learning outcome. Any SAQs that did not meet the requirements of the block objectives, college learning outcomes and the accepted guidelines, taught during the workshop, were corrected or removed. Our investigation estimated the quality of pre-workshop (the academic year 2017–2018) and post-workshop (the academic year 2018–2019) SAQs for Musculoskeletal and Renal courses. A flow-chart for the “well-structured SAQs items’ writing training workshop program” has been given as Fig 1. The study’s primary outcome measures were the outcomes of the final SAQ test items (SAQs for MSK block = 22 pre-workshop and 16 post-workshop, SAQs for Renal block = 18 in the pre-workshop and 17 in the post-workshop) for two successive academic years 2017–2018 (Pre-workshop) and 2018–2019 (post-workshop). The post exam analysis for the said blocks, was measured in terms of items analysis, discriminating index (DI), difficulty factor (P), student’s performance (average mean score and overall passing rate), and reliability of the tests (Kuder-Richardson = Kr-20).

### Quality assessment of the SAQs items

The quality appraisal for the newly constructed SAQs was carried out utilizing, the Kirkpatrick’s model, which offers a helpful assessment structure for the faculty development workshops program. The evaluation model of the Kirkpatrick has been viewed as exceptionally supportive in assessing the workshops with more significant level results [15, 19, 20]. The current study lies in the fourth phase of the Kirkpatrick model, which estimated the improvement in the output of the training workshop for the participants in developing quality SAQs.

### SAQs items analysis in terms of difficulty index, discrimination index, Bloom’s cognitive level, Item writing flaws and Kr-20

The cognitive domain was divided by Bloom’s taxonomy into six progressively organized classifications: knowledge, comprehension, application, analysis, synthesis, and evaluation [20]. The taxonomy was simplified by Tarrant et al., in 2006 and establishing two separate levels, i.e. K1, which reflects basic information knowledge and understanding; K2, which involves implementation and study or recall of the knowledge [21]. K2 level assessment items are stronger, more relevant and differentiate between successful students and low-performing students [15].

Those who violate the norm indicated by item-writing guidelines [22, 23] were the SAQs with item writing flaws. A list of guidelines (in view of the agreement by participants, during the FD workshop) for assessing the consistency of the SAQs was prepared to assess the effectiveness of the FDPs. (S1 Appendix)

In the assessment difficulty index is also named as a P-value or facility index describes as how much percentage of students correctly answered a given test item in an examination. This rang goes from 0 to 100% or 0 to 1. Normally for any easy SAQs test item in the examination has a higher difficulty index and the range or cut-off values between 100% to 70% (easy), similarly 69% to 20% (moderate test items), and 19% to 0% is considered as a difficult test item [22, 24]. However, moderate test items in any assessment (20–69%) have a better discriminating ability between good students and bad students [15]. In any assessment discriminating index is the capacity of a test item to discriminate among high and low achiever students in the particular exam. Higher discriminating indices in any test shows the better and greater discriminating capability of that assessment. The range and the cut-off values for discriminating index > 0.15 is linked with a good test items, and non-discriminating index≤ 0.15 with an average or poor test item [25].

The internal quality and reliability of an exam are calculated by Kuder-Richardson Formula 20 (Kr-20). The KR20 formula for exams with dichotomous choices is a test of internal consistency. If the coefficient of Kr-20 is high (for example, 0.90), this is an indicator of a homogeneous test. If the Kr-20 figure is 0.8, the minimum acceptable value is considered, whereas any figure below 0.8 shows that the exam is not reliable [26].

### Statistical analysis

Data collected was entered in the MSExcel file and analyzed using SPSS (version 21.0) software. To measure and calculate the correlation, Pearson’s chi-square test was used. During the entire study, the statistical significance level was identified as p-value 0.05.

## Results

The SAQs contribute 20% out of 100 marks for Renal and MSK block. The after-effects of the last SAQs based assessment of all two courses (MSK, Renal) for the year 2017–2018 (before workshop training) and 2018–2019 (after workshop training) were dissected independently. The reliability (quality) coefficient (Kr-20) of each of the two courses examined before and after the workshop was more than 0.84. A good improvement was noted for the undergraduate medical students’ average mean score (of the two-course) before workshop training (i.e. 12.51) and after workshop training (i.e.13.67) (Table 1). Also, the general passing rate of students expanded from 67.21 to 73.43% after the SAQ FDPs workshop training. The difficulty index (P) and discrimination index (DI) estimations of the last SAQs based assessments of all the two courses for the pre-workshop training (years 2017–2018) and post-workshop training (academic year 2018–2019) were determined. In the difficulty index over-all moderated questions are improved after the workshop training (i.e.50 to 54.5%). Moreover, the number of hard questions also declined after the workshop training.

**Table 1:** Specification of examination (Total marks = 30).

Courses	Students passed n(%)	Students failed n(%)	Mean score	Standard Deviation	Reliability coefficient
**Pre-workshop**					
MSK Block	185/316 (58.54)	131/316 (41.45)	10.89	4.06	0.86
Renal Block	233/307 (75.89)	74/307 (24.10)	14.13	4.23	0.89
**Post-workshop**					
MSK Block	226/325 (69.53)	99/325 (30.46)	13.22	4.81	0.84
Renal Block	239/309 (77.34)	70/309 (22.65)	14.11	4.44	0.89

The overall result about discrimination index and Bloom’s level showed significant improvement of P-value (X^2^ = 7.32, p = 0.04); (X^2^ = 6.71, p = 0.05), however, difficulty index improvement (X^2^ = 4.69, p = 0.07) was obtained through SAQs for the academic year 2017–2018 and the academic year 2018–2019 for all two courses (Table 2).

**Table 2:** Multiple factors linked to the item analysis.

		MSK		Renal		All two courses		
		Pre*	Post**	Pre*	Post**	Pre*	Post**	
Factors	Categories	n(%)	n(%)	n(%)	n(%)	n(%)	n(%)	x2(P)
Difficulty Index	Easy (>70%)	8/22(36.36)	6/16(37.5)	8/18(44.4)	9/17(52.9)	16(40)	15(45.4)	4.69(0.07)
	Moderate (20–70%)	11/22 (50)	10/16(66.67)	9/18(50)	8/17(47.0)	20(50)	18(54.5)	
	Difficult (<20%)	3/22(13.6)	0/16(0.00)	1/18(5.5)	0/17(100)	4(10)	0(0.00)	
	Total	22(100)	16(100)	18(100)	17(100)	40(100)	33(100)	
Discrimination Index	DI(>0.15)	16/22(72.7)	12/16(75)	11/18(61.1)	13/17(76.47)	27(67.5)	25(75.7)	7.32(0.04)
	Non-Di(≤0.15)	6(27.2)	4/16(25)	7/18(38.8)	4/17(23.52)	13(32.5)	8(24.2)	
	Total	22(100)	16 (100)	18(100)	17(100)	40(100)	33(100)	
Items writing flaws (IWFs)	IWF	4/22 (18.1)	1/16 (6.25)	1/18(5.56)	0/17(0)	5 (12.5)	1 (3.0)	1.09(0.39)
	Without-IWF	18/22(81.8)	15/16(93.7)	17/18(94.4)	17/17(100)	35(87.5)	32 (96.9)	
	Total	22(100)	16(100)	18(100)	17(100)	40(100)	33(100)	
Bloom’s taxonomy levels	K1	15(68.1)	9(56.2)	13(72.2)	9(52.94)	28(70)	18(54.5)	6.71(0.05)
	K2	7(31.8)	7(43.7)	5(27.7)	8(47.05)	12(30)	15(45.5)	

*Pre-workshop training

** Post-workshop training, K1- Non-scenario based question, K2- scenario-based questions

The flawed questions in the exam were also found to be less in number, after the post-workshop training 5/40 (12.5%) to 1/33 (3.0%) in the year 2018–2019. The SAQs were further divided into easy, moderate and difficult categories based on their difficulty level (Table 2). The hard or difficult questions reduced, and percentage of moderated questions was increased from 50% to 54.5% in the academic year 2018–2019 (post-workshop training) (Table 2).

In the Bloom’s cognitive level in current study, during the academic year 2017–2018 (pre-workshop training) the K1 questions were more than the K2 questions in both blocks, but after workshop training the number of K2 questions increased 12(30%) to 15 (45.5%) in the academic year 2017–2018 and K1 questions reduced 28(70%) to 18 (54.5%).

## Discussion

FDPs are essentially required to prepare faculty members in pursuit of the positive changeover towards quality assessment.,. Initially, FDPs were conceptualized as methods for enhancing the efficiency of teaching & learning environment [27–29]. In order to cope with the rapid changes, faculty members need to reinforce their abilities from time to time, and in this regard the FDPs act as essential tool [29]. In their academic duties, many untrained educationists perform well, but earlier results showed that they could be more effective with formal training in their roles [30]. Moreover, professional development is established by FDPs, especially for new faculty members to help them succeed in their many vital academic roles. Therefore, FDPs have become very relevant, and convincing in terms of faculty’ improvements in learning, behavior and performance [1]. In the current study the results confirmed the usefulness of FDP by means of SAQs item’s writing workshop, with enhancement in positive items-related outcomes, as well as an improvement in student’s marks (mean score) with higher passing rate. The results analysis also showed a substantial positive difference in the calculated outcome, including the difficulty index and the discrimination index of the final SAQs-based exams of two individual courses in 2018–2019 (post-workshop) as compared with over the 2017–2018 (pre-workshop). The current research indicates that the discrimination index (DI-values) resulted in substantial changes in the consistency of the production of test items by the new faculty participants with FDPs in medical academics. In pre-and post-workshop faculty training review, generous contrasts were seen for DIs, as a good number of SAQs were available in both the two courses and exhibited substantial progress after FDPs training. The consistency of the SAQs assessment items by the participants was also showed to be improved after attending the FDPs. Overall improvement in construction of SAQs items’ writing skills in post-workshop training phase reflected expanded mean score of the assessment and higher passing frequency of students. In the current study our results were accorded with the previous work [1, 22]. Moreover, while assessing the impact of FDPs on quality of MCQs, short answer questions (SAQ) and objective structured clinical examination (OSCE) items writing, we found a noteworthy upgrading [1]. Similarly, there is a strong impact of dedicated FDPs on improving the quality of the review items [1]. Jozefowicz et. Al. at 2002 stated that the United State Medical Licensing Examination (USMLE) items prepared by skilled faculties had a higher mean student score versus the items prepared faculties without proper training [31]. Peer review and formal training both are shown to enhance the standard of item writing [32, 33]. The current study specified that the pre-and post-workshop training reliability (Kr-20) of the examination was very good ≥0.84 but not excellent depending on the number of SAQs questions produced. This might be due to the fact that test reliability doesn’t only rest on the quality of the SAQs but also ‘the number of SAQs’, ‘distribution of the grades’ and the ‘time provided for the examination’ play important role [34]. The current study findings indicate that the FDPs enhance the evaluation which in turn derives learning [35, 36]. Problematic questions or item writing defects (IWFs) are among the issues with the stability of any evaluation items. Post workshop instructions and training helped get rid of such issues in the present report. Many researchers have acknowledged possible explanations for the deficiency of quality of test items, and IWF’s was one of the significant causes [37, 38]. Vyas and Supe have stated that the lack of proper and focused training in the field of MCQs and SAQs construction, causes more flaws in writing quality items [37]. A similar finding was found our results also decreased the IWFs after the FDPs, the passing rate is increased. IWFs also have an impact on the index of discrimination and the index of difficulties as poor discrimination and low difficulty support low scorers. Moreover, low discrimination and high difficulty indices have a negative impact on high scorers [39]. The objective of course learning also influences flawed assessment items [39]. The excellent means of assessment motivate and encourage students’ attitude towards learning. The current study highlights that the faculty should be encouraged and trained to construct SAQs for cognitive levels of higher quality to properly evaluate trainees [40]. The current study also found that faculty development program helps improve the quality and validity of the examination item i.e. SAQs as well as the deep learning tactics of students. The results specify that, in teaching organizations FDPs should be arranged regularly. This research recommends that freshly joined faculty members attend and actively participate in all future FDPs (specifically targeting for assessment tools such as OSCE, OSPE, SAQs and even more topics depending on the exam structure of medical college) on a regular basis. As a reference, a flow chart of the writing training workshop program structure of MCQs items was provided according to the evaluation levels of Kirkpatrick (Fig 1).

## Conclusion

Training improves faculty’s assessment skills leading to an overall improvement in the quality of the SAQs with an overall reduction for item writing flaws. Additionally, a proper follow-up process for assessing quality of SAQs in coordination with involved faculty will bring a lasting improvement of assessment.

## Supporting information

S1 Appendix (DOCX)

S1 Glossary (DOCX)
