# Supplementary material for: Faculty development program assists the new faculty in constructing high-quality short answer questions; a quasi-experimental study
Source: PLoS One. 2021 Mar 29;16(3):e0249319. doi: 10.1371/journal.pone.0249319 (PMC8007032; doi:10.1371/journal.pone.0249319)
Supplement: S1 Appendix — (DOCX) [file pone.0249319.s001.docx]

**APPENDIX-1**

**Short Answer Question Guidelines (SAQ), College of Medicine, King Saud University.**

1. Set learning objectives first.

- Make sure that SAQs are aligned with specified learning objectives.
- Make sure that SAQs are aligned with college learning outcomes.

1. SAQs are representative of the taught content and relevant for the purpose of the exam.
2. Students can understand as what exactly is expected of them for each question/sub-question.

- Questions are straight forward.
- Questions/sub-questions focus on single aspect and or piece of knowledge.
- Questions are written in easy to understand and in an unambiguous manner.
- Questions are not too short/too long. .

1. 60 to 70 % of SAQs questions should be scenario-based and the scenario should be functionally related to the question.
2. Define the key words for checking.
3. There should not be a ‘word’ or any sentence in the scenario, so that correct answer could be guessed.
4. The terms such as 'most likely' and 'best' are utilized when essential.
5. There is a limit specified for the length of answers whenever required.
6. Questions must have marks mentioned.

- The mark allocation reflects the importance of each question.

1. Categorize your SAQ questions according to the exam- blueprint.
