# Supplementary material for: Faculty development program assists the new faculty in constructing high-quality short answer questions; a quasi-experimental study
Source: PLoS One. 2021 Mar 29;16(3):e0249319. doi: 10.1371/journal.pone.0249319 (PMC8007032; doi:10.1371/journal.pone.0249319)
Supplement: S1 Glossary — (DOCX) [file pone.0249319.s002.docx]

AEC = Assessment and evaluation center

COM = Collge of Medicine

DI = Discriminatory Index

DF = Difficulty Factor

FDPs = Faculty Development Programs

KSU = King Saud University

Kr= Kuder-Richardson

MCQ = Multiple Choice Question

MSK = Musculoskeletal

OSPE = Objective Structured Practical Examination

OSCE = Objective Structured Clinical Examination

SAQ = Short Answer Question
